# Vascular Protective Effect of an Ethanol Extract of *Camellia japonica* Fruit: Endothelium-Dependent Relaxation of Coronary Artery and Reduction of Smooth Muscle Cell Migration

**DOI:** 10.1155/2016/6309565

**Published:** 2015-11-30

**Authors:** Sin-Hee Park, Bong-Sup Shim, Jun-Seong Yoon, Hyun-Ho Lee, Hye-Won Lee, Seok-Bong Yoo, An-Jin Wi, Whoa-Shig Park, Hyun-Jung Kim, Dong-Wok Kim, Min-Ho Oak

**Affiliations:** ^1^College of Pharmacy and Natural Medicine Research Institute, Mokpo National University, Muan-gun, Jeonnam 58554, Republic of Korea; ^2^Jeonnam Forest Resources Research Institute, Naju, Jeonnam 58213, Republic of Korea; ^3^Department of Oriental Medicine Resources, Mokpo National University, Muan-gun, Jeonnam 58554, Republic of Korea

## Abstract

*Camellia japonica* is a popular garden plant in Asia and widely used as cosmetic sources and traditional medicine. However, the possibility that *C. japonica* affects cardiovascular system remains unclear. The aim of the present study was to evaluate vascular effects of an extract of *C. japonica*. Vascular reactivity was assessed in organ baths using porcine coronary arteries and inhibition of proliferation and migration were assessed using human vascular smooth muscle cells (VSMCs). All four different parts, leaf, stem, flower, and fruits, caused concentration-dependent relaxations and *C. japonica* fruit (CJF) extract showed the strongest vasorelaxation and its effect was endothelium dependent. Relaxations to CJF were markedly reduced by inhibitor of endothelial nitric oxide synthase (eNOS) and inhibitor of PI3-kinase, but not affected by inhibitor of cyclooxygenase and endothelium-derived hyperpolarizing factor-mediated response. CJF induced activated a time- and concentration-dependent phosphorylation of eNOS in endothelial cells. Altogether, these studies have demonstrated that CJF is a potent endothelium-dependent vasodilator and this effect was involved in, at least in part, PI3K-eNOS-NO pathway. Moreover, CJF attenuated TNF-*α* induced proliferation and PDGF-BB induced migration of VSMCs. The present findings indicate that CJF could be a valuable candidate of herbal medicine for cardiovascular diseases associated with endothelial dysfunction and atherosclerosis.

## 1. Introduction

Across the world, cardiovascular diseases (CVDs) are the primary cause of deaths. In the past, CVDs were considered to be diseases of developed countries. However, recently, CVDs have become most notable in developing countries and this reflects meaningful global changes in behavior and lifestyle [1]. Endothelial dysfunction is a major pathological condition which is almost associated with a risk marker for cardiovascular diseases such as atherosclerosis [2]. Endothelial cells have a key role in the control of vascular homeostasis in part via the release of potent vasodilators such as nitric oxide (NO) and excessive cell proliferation of vascular smooth muscle cells is a key contributor to the growth of atherosclerotic plaque and restenosis.

Several epidemiological studies have been reported that regular intake of plants-derived food such as tea, cacao, and red wine is associated with a reduced risk of cardiovascular diseases [3–7]. The protective effect has been attributed, at least in part, to their high polyphenol content, which might protect the cardiovascular system by a variety of actions, including the ability to dilate blood vessels by stimulating the endothelial formation of NO [8] and to inhibit proliferation and migration of vascular smooth muscle cells [8, 9]. Taken together, these findings suggest the view that polyphenol-rich natural products may protect the cardiovascular system, in part, by improving the vascular homeostasis, hence, retarding the development of cardiovascular diseases.

In order to identify vasoprotective natural products, we have recently evaluated the cardiovascular protective properties of medicinal plants extracts used in oriental medicine on isolated porcine coronary artery and human vascular smooth muscle cells (VSMCs). These investigations have revealed a strong cardiovascular protective effect of an ethanolic extract of* Camellia japonica* fruits (CJFs).

Previous studies have shown that* C. japonica* (CJ), extensively distributed in Japan and Korea, possesses various biological activities, including antioxidant activity [10, 11], antimetastasis activity [12], antiallergic responses [13], and antibacterial activity [14]. However, these findings have been studied in oil, flower, or leaf of CJ. As constituents of CJ, saponins in the seeds [15], flavonol glycosides in the leaves [10], and triterpenes, several hydrolyzable tannins, acylated anthocyanins, and purine alkaloids in the flowers [16] have been reported. But the chemical constituents and pharmacological activity of fruits have been reported.

The aim of the present study was to evaluate the vasoprotective effect of an extract of* C. japonica* fruits on vascular functions, if so, to characterize the underlying mechanism and signaling pathway involved. In particular, we have determined the ability of CJF (1) to cause endothelium-dependent relaxation of porcine coronary artery, (2) to activate endothelial NO synthase by phosphorylation in cultured endothelial cells, and (3) to prevent proliferation and migration of vascular smooth muscle cells induced by growth factors.

## 2. Materials and Methods

### 2.1. Plant Extract

The* C. japonica* (CJ) was collected at the southern parts of Korean Peninsula and voucher specimen was deposited at the Herbarium of Jeonnam Forest Resources Research Institute, Korea. Each dried leaf, stem, fruit, and flower was cut into small pieces and ground using a commercial food mixer. The fruits of CJ (1 Kg) were extracted two times with hot 70% ethanol for 4 hours. This residue was evaporated in vacuo to yield the total extract (93.4 g, 9.32% w/w). A solution was prepared with physiological salt solution (PSS) at all concentration of 100–300 mg/mL on the day of the experiment.

### 2.2. Vascular Reactivity Study

Vascular reactivity study was performed using porcine coronary arteries as described previously [17]. Briefly, left anterior descending coronary arteries of porcine heart (got from the local slaughterhouse in Mokpo, Korea) were dissected and cleaned of connective tissue and cut into rings (4-5 mm in length) carefully. Then, rings were suspended in organ baths containing oxygenated (95% O_2_ and 5% CO_2_) Krebs bicarbonate solution (mmol/L: NaCl 119, KCl 4.7, KH_2_PO_4_ 1.18, MgSO_4_ 1.18, CaCl_2_ 1.25, NaHCO_3_ 25, and D-glucose 11, pH 7.4, 37°C) for the determination of changes in isometric tension. Following equilibration for 90 min under a resting tension of 5 g, rings were twice contracted with KCl (80 mmol/L). Thereafter, the rings were precontracted with the thromboxane mimetic U46619 (1–60 nmol/L) to about 80% of the maximal contraction and the relaxation to bradykinin (0.3 *μ*mol/L) was determined. After washout and a 30-minute equilibration period, rings were again contracted with U46619 before a concentration-relaxation curve to plant extract. In some experiments, rings were exposed to an inhibitor for 30 min before the addition of U46619.

### 2.3. Cell Culture

Endothelial cells were isolated from porcine left anterior descending coronary arteries by collagenase treatment (type I, Worthington, 1 mg/mL for 12 min at 37°C) and cultured in T-flasks containing medium MCDB 131 supplemented with 15% fetal bovine serum (FBS), penicillin (100 U/mL), streptomycin (100 U/mL), fungizone (250 *μ*g/mL), and L-glutamine (2 mM) and grown for 48–72 h. Confluent cultures of cells (first passage) were exposed to serum-free culture medium in the presence of 0.1% bovine serum albumin for 6 h prior to treatment. Vascular smooth muscle cells (VSMCs) were purchased from Bio-Whittaker (San Diego, CA) and cultured in MCDB131 with 10% FBS and antibiotics. For all experiments, early passages of VSMCs were grown to 80–90% confluence. In a typical experiment, the cells were starved in serum-free culture medium containing 0.1% bovine serum albumin for 24 h.

### 2.4. Determination of the Phosphorylation Level of eNOS

Endothelial cells were lysed in extraction buffer. Total proteins (10 *μ*g) were separated on SDS-polyacrylamide gels at 100 V for 2 h. Separated proteins were transferred onto polyvinylidene difluoride membranes at 100 V for 2 h. Membranes were blocked with bovine serum albumin for 1 h. Membranes were incubated with a primary antibody, p-eNOS Ser1177 from rabbit. After washing, membranes were incubated with the appropriate horseradish peroxidase-conjugated secondary antibody. The blots were then washed 5-6 times in TBST and developed using an enhanced chemiluminescence (ECL) detection kit.

### 2.5. Proliferation Assay

VSMCs proliferation was assessed by using MTT assay. VSMCs seeded in 96-well plates were pretreated with CJF for 1 h prior to stimulation of the cells with TNF-*α* (100 *μ*g/mL) for additional 24 h. Then, MTT solution was added for 4 h followed by solubilization of formazan crystals in dimethyl sulfoxide (DMSO). The purple color thus formed was measured at 540 nm.

### 2.6. Cell Migration Assay

VSMCs were assessed in wound healing scratch assays using the IncuCyte (Essen Bioscience). Briefly, 4 × 10^5^ cells were plated on the 96-well ImageLock plates (Essen BioScience, catalog number 4379) and incubated in the complete media until confluent monolayer forms. Wounds were made using the 96-pin WoundMaker (Essen BioScience) and incubated with MCDB131 containing 0.1% BSA and PDGF-BB (20 ng/mL) with or without increasing concentrations of CJF. Cell migration was monitored in real time by IncuCyte, and confluence was measured by the IncuCyte software.

### 2.7. Statistical Analysis

Values are expressed as means ± SEM. Statistical evaluation was performed with Student's *t*-test for paired data or ANOVA followed by Fisher's protected least significant difference test where appropriate. ED_50_ is defined as the concentration of CJ extracts causing 50% relaxation. Values of *P* < 0.05 were considered statistically significant.

## 3. Results

### 3.1. CJF Induces Endothelium-Dependent Relaxation in Porcine Coronary Arteries

**Figure 1:**
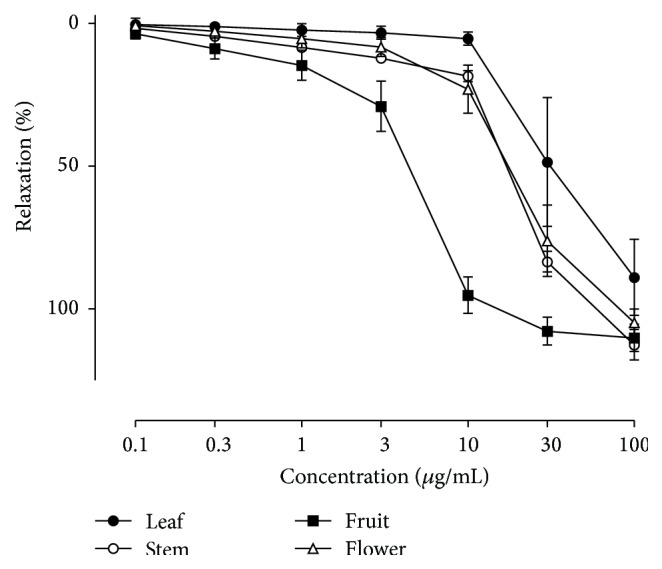
Extracts from several different parts of* C. japonica* cause endothelium-dependent relaxations in the porcine coronary artery. Arterial rings with endothelium were contracted with U46619 before the addition of increasing concentrations of either leaf, stem, fruit, or flower of* C. japonica*. The relaxation response is expressed as the percentage relaxation of the U46619-induced contraction. Results are shown as means ± SEM (*n* = 6).

**Figure 2:**
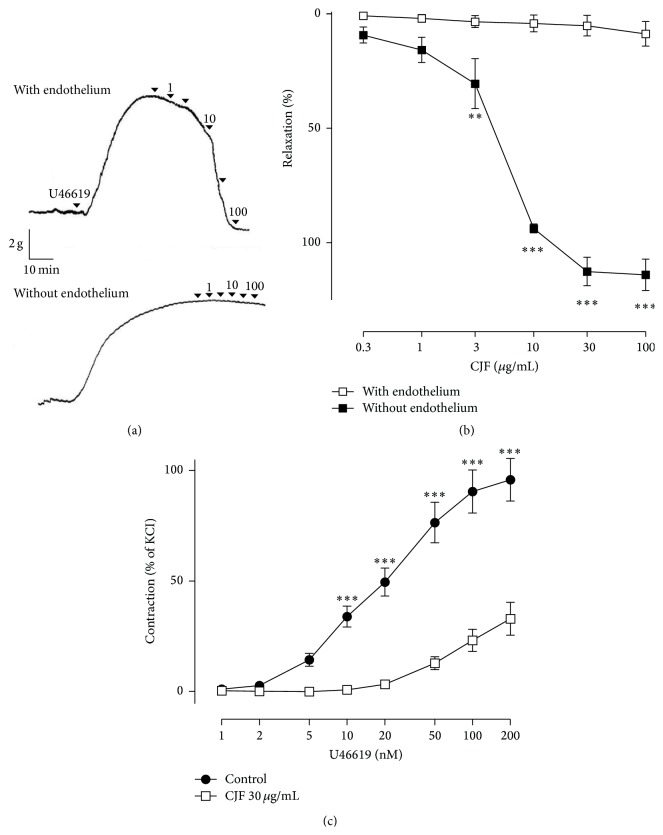
Characterization of endothelium-dependent relaxations to CJF in porcine coronary artery rings. Representative original tracing (a), corresponding cumulative data (b). The relaxation response is expressed as the percentage relaxation of the U46619-induced contraction. Effect of CJF on contractile responses (c). As indicated, intact rings were exposed to CJF (30 *μ*g/mL) 30 min before the addition of increasing concentrations of U46619. Results are shown as means ± SEM (*n* = 7). ^*∗*^ *P* < 0.05, ^*∗∗*^ *P* < 0.01, ^*∗∗∗*^ *P* < 0.001, significant difference versus rings without endothelium (b) or control (c).

CJ extracts from four different parts, leaf, stem, fruit, and flower, were evaluated for their potency of concentration-dependent relaxations of porcine coronary artery rings (Figure 1). All four different parts caused concentration-dependent relaxations and* C. japonica* fruit extract (CJF) is the most active one. The ED_50_ value of the vasorelaxing effect of leaf, stem, flower, and fruit extracts was 88.70 ± 2.29 *μ*g/mL, 38.24 ± 1.31 *μ*g/mL, 33.89 ± 1.39 *μ*g/mL, and 5.37 ± 1.33 *μ*g/mL, respectively. Although CJF caused full relaxations in porcine coronary artery rings with endothelium, no such effect was observed in those without endothelium indicating that CJF induced endothelium-dependent vasorelaxations (Figures 2(a) and 2(b)). Next, the possibility that CJF, besides inducing endothelium-dependent relaxations, also affects contractile responses was assessed. Exposure of porcine coronary artery rings to CJF concentration-dependently reduced contractile responses to thromboxane mimetic U46619 in rings (Figure 2(c)).

### 3.2. CJF Induces Endothelium-Dependent NO-Medicated Relaxation via the Redox-Sensitive PI3-Kinase Pathway

**Figure 3:**
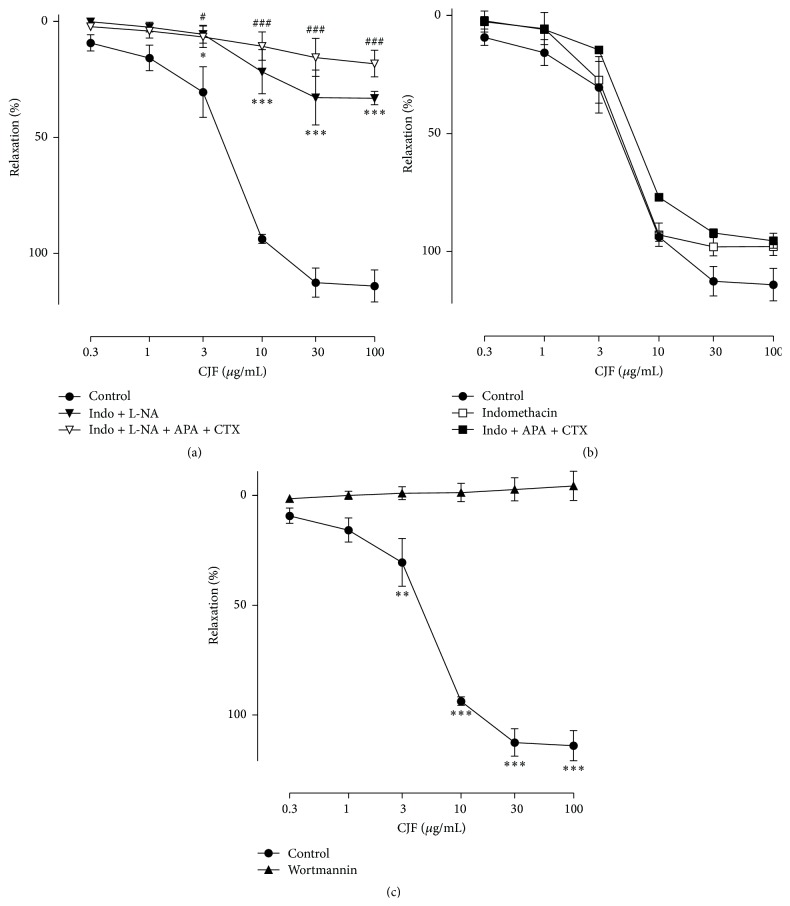
Characterization of endothelium-dependent relaxations to CJF in porcine coronary artery rings. Rings with endothelium were incubated with either indomethacin (10 *μ*M), N^*ω*^-nitro-L-arginine (L-NA, 10 *μ*M), charybdotoxin (CTX, 100 nM) plus apamin (APA, 100 nM), or the PI3-kinase inhibitors wortmannin (30 nM) for 30 min before the contraction to U46619 and subsequent relaxation to CJF. The relaxation response is expressed as the percentage relaxation of the U46619-induced contraction. Results are shown as means ± SEM (*n* = 5). ^*∗*^ *P* < 0.05, ^*∗∗*^ *P* < 0.01, ^*∗∗∗*^ *P* < 0.001, significant difference versus control.

Since previous investigations have shown that plants-derived polyphenols induce the redox-sensitive PI3-kinase/Akt-dependent activation of endothelial NO synthase to cause vasorelaxation [18], the role of this pathway in the CJF-induced relaxation was determined. Further characterization of the endothelium-dependent relaxation was done with CJF. Relaxations to CJF in rings with endothelium were markedly reduced by N^*ω*^-nitro-L-arginine (L-NA, endothelial nitric oxide synthase inhibitor) but not minimally modified by indomethacin (an inhibitor of cyclooxygenases) and the combination of charybdotoxin plus apamin (inhibitors of EDHF-mediated responses, Figures 3(a) and 3(b)). In addition, the combination of L-NA plus charybdotoxin and apamin abolished relaxations to CJF, suggesting the involvement of NO-mediated component and also, to some extent, an EDHF-mediated component (Figure 3(a)). Relaxations to CJF also strongly reduced by the PI3-kinase inhibitor, wortmannin (Figure 3(c)).

### 3.3. CJF Induces the Phosphorylation of eNOS at Ser1177 in Endothelial Cells

**Figure 4:**
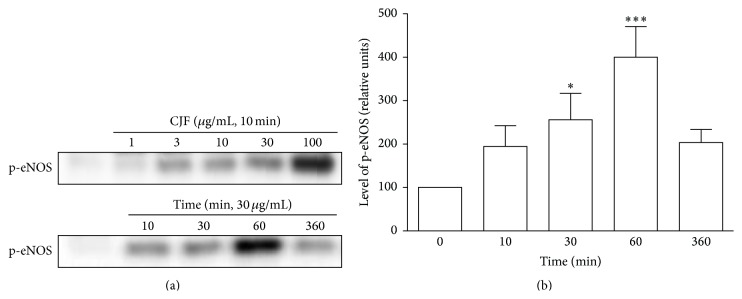
CJF induces a concentration- and time-dependent phosphorylation of eNOS at Ser1177 in endothelial cells. Cells were exposed to CJF for the indicated concentrations and times at 37°C. Thereafter, the level of p-eNOS was determined by Western blot analysis. (a) Representative immunoblots and (b) corresponding cumulative data for time-dependent phosphorylation of eNOS. Results are shown as means ± SEM (*n* = 3-4). ^*∗*^ *P* < 0.05, ^*∗∗*^ *P* < 0.01, ^*∗∗∗*^ *P* < 0.001, significant difference versus control.

To better characterize the signaling pathway involved in eNOS activation in response to CJF, level of phosphorylated eNOS was assessed in endothelial cells by immunoblotting. Unstimulated endothelial cells had either no or only a low level of phosphorylated eNOS at ser1177. CJF (1–100 *μ*g/mL, 10 min) evoked the concentration-dependent phosphorylation of eNOS at ser1177 in endothelial cells (Figure 4(a)). Exposure of endothelial cells to CJF (30 *μ*g/mL) caused the progressive appearance of strong phosphorylation signal of eNOS up to 60 min (Figures 4(a) and 4(b)).

### 3.4. CJF Inhibits VSMC Proliferation and Migration

**Figure 5:**
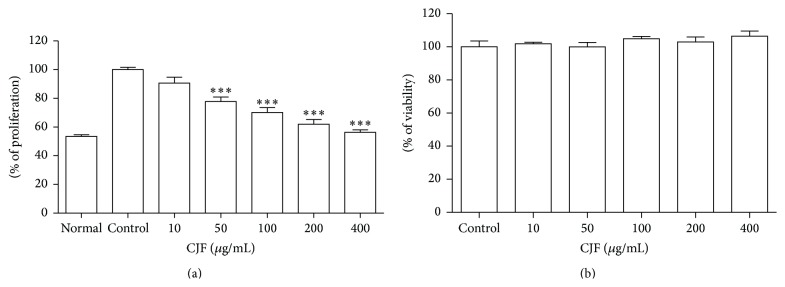
(a) Serum starved VSMCs were pretreated with indicated concentrations of CJF followed by stimulation with TNF-*α* (100 *μ*g/mL) for 24 h. Cell proliferation was measured by MTT assay and values are expressed as means ± SEM (*n* = 5). ^*∗*^ *P* < 0.05, ^*∗∗*^ *P* < 0.01, ^*∗∗∗*^ *P* < 0.001, significant difference versus control. (b) Serum starved VSMCs were treated with indicated concentrations of CJF without stimulation for 24 h. Cell viability was measured by MTT assay. Results are shown as means ± SEM (*n* = 5). ^*∗*^ *P* < 0.05, significant difference versus control.

To characterize cardiovascular protective effect of CJF, we have performed experiments whether CJF inhibits proliferation and migration of VSMCs. Stimulation of VSMCs with TNF-*α* induced proliferation and the treatment of CJF concentration-dependently inhibited VSMCs proliferation induced by TNF-*α* (Figure 5(a)). CJF at concentrations 50, 100, 200, and 400 *μ*g/mL significantly reduced the proliferation rate to 77.85%, 70.12%, 61.93%, and 56.33% of the control (TNF-*α* treated without CJF), respectively. To confirm that the inhibitory effects were not due to toxicity or damage to the cells, various concentrations of CJF were treated in nonstimulated cells for 24 h. CJF had no effect on the basal level of cell viability (Figure 5(b)).

**Figure 6:**
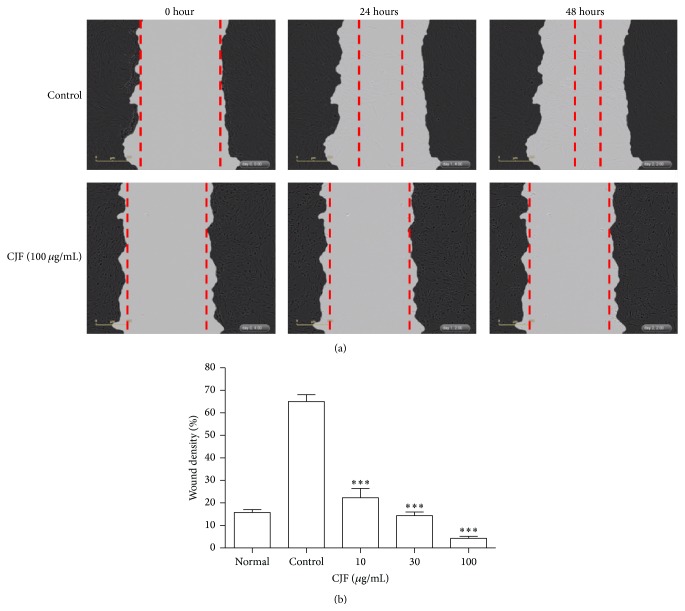
(a) Confluent VSMCs were gently removed using the 96-pin WoundMaker to induce reproducible wounds and then the cells were treated with CJF for 1 h followed by PDGF-BB treatment. Images of wounded area were captured immediately (time 0) and 24 and 48 h after injury. (b) Finally, wound density was quantified as percentage of initial wound area that had been recovered with VSMCs. Results are shown as means ± SEM (*n* = 5). ^*∗*^ *P* < 0.05, ^*∗∗*^ *P* < 0.01, ^*∗∗∗*^ *P* < 0.001, significant difference versus control (PDGF-BB alone).

The effects of CJF on VSMCs migration as evaluated by using a wound healing assay are as shown in Figure 6. CJF concentration-dependently suppressed PDGF-BB induced VSMCs wound healing for 24 and 48 h after injury (Figure 6(b)). CJF at 10, 30, and 100 *μ*g/mL showed the significant inhibition of healing (22.28%, 14.38%, and 4.3% compared with control, resp.) at 48 h.

## 4. Discussion

Cardiovascular diseases (CVDs) such as atherosclerosis are associated with endothelial dysfunction, and these CVDs risk factor modification leads to improvement in vascular function. Endothelial dysfunction is a pathological condition, mostly characterized by an imbalance between vasodilator and vasoconstrictor substances, and this imbalance leads to an impairment of endothelium-dependent relaxation, which appears the functional characteristic of endothelial dysfunction. Endothelial cells lining the luminal surface of all blood vessels have a vital role in the control of vascular tone partially via the release of powerful vasodilators including nitric oxide (NO) and endothelium-derived hyperpolarizing factor (EDHF). Particularly, the endothelium-dependent relaxation was involved in increased guanosine 3′, 5′-cyclic monophosphate (cyclic GMP) levels in the intact aorta and both the relaxation and the increase in cyclic GMP were blocked by an inhibition of eNOS synthase [19]. In addition, NO inhibits other major events in the development of atherosclerosis such as platelet aggregation and smooth muscle cell proliferation [2, 20]. Especially in the microcirculation, prostacyclin and endothelial-derived hyperpolarization factors (EDHF) also play a key role [21].

Among the several different parts of* C. japonica* (CJ) investigated in present study, the most active one was predominantly fruit of CJ. The present investigations indicate that CJF is a potent endothelium-dependent vasodilator of coronary arteries and that this activity involves several elements. Endothelium-dependent relaxations to CJF were markedly inhibited by L-NA, an endothelial NO synthase inhibition. In contrast, these relaxations were not affected by indomethacin and the combination of charybdotoxin plus apamin, ruling out the involvement of vasoactive prostanoids and EDHF. In addition to inducing relaxation, CJF also significantly blunted contractile response to vasoconstrictors such as thromboxane analogue, U46619. Since no such effects are observed in rings without endothelium and the beneficial effect of the endothelium is abolished by N^*ω*^-nitro-L-arginine, the blunted contractile responses are most likely due to the ability of CJF to stimulate the endothelial formation of NO.

Previous studies have indicated that the PI3-kinase pathway mediates the increases of eNOS activation and NO formation in response to several stimuli, including shear stress, and polyphenols through the PI3K-Akt-dependent phosphorylation of eNOS at Ser1177, resulting in an increased formation of NO and vasorelaxation [18, 22]. Indeed, our findings indicated a rapid increase of phosphorylation of eNOS at Ser1177 in concentration-dependent and time-dependent manner in endothelial cells. Moreover, inhibition of PI3-kinase pathway by wortmannin blocked the CJF-induced coronary artery relaxation. Altogether, the present findings indicate that CJF causes powerful endothelium-dependent relaxations involving, at least in part, PI3-kinase-eNOS-NO pathway.

Additionally, we performed other experiments under a different aspect. Since VSMCs proliferation and migration are one of the leading factors in the progression of atherosclerosis [23], inhibition of VSMCs proliferation and migration represents a critical therapeutic strategy for the prevention of atherosclerosis. Various growth factors including PDGF-BB and TNF-*α* are upregulated in atherosclerosis [24, 25]. In our* in vitro* model of VSMCs proliferation, the treatment of CJF concentration-dependently inhibited TNF-*α* induced VSMCs proliferation. At least, VSMCs migration would require a chemoattractant to direct their movement toward the intima, the ability to breach and transverse the ECM barriers, and the activation of the cellular machinery for cell movement in response to the chemoattractant [26]. Our studies used PDGF-BB as a chemoattractant, since considerable evidence supports a role of PDGF-BB in VSMCs migration. CJF strongly inhibited PDGF-BB induced VSMCs migration.

*Camellia japonica* (“Dongbaek” in Korean) is a popular garden plant in Korea. In addition, flower, seed, and leaves are widely used as cosmetic sources and traditional medicine. Previous study revealed that extracts from different parts of* C. japonica* have various biological activities such as antioxidant activity [10, 11], antimetastasis activity [12], antiallergic responses [13], and antibacterial activity [14]. However, these biological activities are focused on seeds, flowers, and leaves, not fruits. Until now, the activities of fruit from* C. japonica* have drawn much less attention than those of other parts from* C. japonica*. The present findings indicated that fruit from* C. japonica* has strong cardiovascular protection effects and could be a good candidate of natural medicines for prevention and treatment of cardiovascular diseases. Despite these beneficial effects from fruit, very little information regarding its constituents was not available to compare with seed and leaves from* C. japonica*. Very recently, Uddin et al. isolated oleanane-type triterpenes from fruit peels of* C. japonica* as protein tyrosine phosphatase 1B inhibitors [27]. The vasoprotective active compounds from CJF have not been identified yet due to the lack of constituents' study; therefore, we will undertake further study to identify active compounds and their underlying molecular mechanisms.

## 5. Conclusions

The present studies indicate that* Camellia japonica* fruit (CJF) extract is a potent endothelium-dependent vasodilator by inducing the endothelial formation of NO via PI3-kinase pathway in endothelial cells. In addition, CJF attenuated TNF-*α* induced VSMCs proliferation and PDGF-BB induced VSMCs migration. They further suggest that CJF could be a valuable candidate of herbal medicine for cardiovascular diseases associated with endothelial dysfunction and atherosclerosis.
